# 

**Published:** 1902-06-15

**Authors:** 


### CONTENTS.

President's Address, - - *By C. R. Rowley, D.D.S.* 271

The Past and Future in Dentistry, *By F. H. Essig, D.D.S.* 280

Medical Specialties, - *By E. I. Backus, D.D.S.* 283

A Little of Several Things, *By Dr. Charles H. Warboys, D.D.S.* 294

Dental Education in the Public Schools. *By Prof. W. L. Mercer* 300

The Ladies, - - - - *By Dr. B. Le Gro* 308

The Value of Gutta-Percha in Dentistry, *By Dr. Litch* 311

Editorial, - - - - - - - - - - 313

Announcements, - - - - - - - - - 315

Indents, - - - - - - - - - - 316

